# Identification of a novel locus associated with skin colour in African-admixed populations

**DOI:** 10.1038/srep44548

**Published:** 2017-03-16

**Authors:** Natalia Hernandez-Pacheco, Carlos Flores, Santos Alonso, Celeste Eng, Angel C. Y. Mak, Scott Hunstman, Donglei Hu, Marquitta J. White, Sam S. Oh, Kelley Meade, Harold J. Farber, Pedro C. Avila, Denise Serebrisky, Shannon M. Thyne, Emerita Brigino-Buenaventura, William Rodriguez-Cintron, Saunak Sen, Rajesh Kumar, Michael Lenoir, Jose R. Rodriguez-Santana, Esteban G. Burchard, Maria Pino-Yanes

**Affiliations:** 1 Research Unit, Hospital Universitario N.S. de Candelaria, Universidad de La Laguna, Santa Cruz de Tenerife, Spain; 2 Applied Genomics Group (G2A), Section of Genetics, Department of Biochemistry, Microbiology, Cell Biology and Genetics, Universidad de La Laguna, San Cristobal de La Laguna, Tenerife, Spain; 3 CIBER de Enfermedades Respiratorias, Instituto de Salud Carlos III, Madrid, Spain; 4 Department of Genetics, Physical Anthropology and Animal Physiology, University of the Basque Country UPV/EHU, Leioa, Bizkaia, Spain; 5 Department of Medicine, University of California, San Francisco, CA, United States; 6 Children’s Hospital and Research Center Oakland, Oakland, CA, USA; 7 Department of Pediatrics, Section of Pulmonology, Baylor College of Medicine and Texas Children’s Hospital, Houston, TX, USA; 8 Allergy & ENT Associates, Woodlands, TX, USA; 9 Pediatric Pulmonary Division, Jacobi Medical Center, Bronx, NY, USA; 10 Department of Pediatrics, University of California San Francisco, San Francisco General Hospital, San Francisco, CA, USA; 11 Department of Allergy and Immunology, Kaiser Permanente-Vallejo Medical Center, Vallejo, CA, USA.; 12 Veterans Caribbean Health Care System, San Juan, Puerto Rico; 13 Division of Biostatistics, Department of Preventive Medicine, UTHSC, Memphis, TN, USA; 14 Children’s Memorial Hospital and the Feinberg School of Medicine, Northwestern University, Chicago, IL, USA; 15 Bay Area Pediatrics, Oakland, CA, USA; 16 Centro de Neumología Pediátrica, San Juan, Puerto Rico; 17 Department of Bioengineering and Therapeutic Sciences, University of California, San Francisco, CA, United States

## Abstract

Skin pigmentation is a complex trait that varies largely among populations. Most genome-wide association studies of this trait have been performed in Europeans and Asians. We aimed to uncover genes influencing skin colour in African-admixed individuals. We performed a genome-wide association study of melanin levels in 285 Hispanic/Latino individuals from Puerto Rico, analyzing 14 million genetic variants. A total of 82 variants with *p*-value ≤1 × 10^−5^ were followed up in 373 African Americans. Fourteen single nucleotide polymorphisms were replicated, of which nine were associated with skin colour at genome-wide significance in a meta-analysis across the two studies. These results validated the association of two previously known skin pigmentation genes, *SLC24A5* (minimum *p* = 2.62 × 10^−14^, rs1426654) and *SLC45A2* (minimum *p* = 9.71 × 10^−10^, rs16891982), and revealed the intergenic region of *BEND7* and *PRPF18* as a novel locus associated with this trait (minimum *p* = 4.58 × 10^−9^, rs6602666). The most significant variant within this region is common among African-descent populations but not among Europeans or Native Americans. Our findings support the advantages of analyzing African-admixed populations to discover new genes influencing skin pigmentation.

Skin pigmentation is essential in the protection against ultraviolet (UV) radiation^1^,^2^. A complex regulatory system controls the production of melanin^3^, the main pigment providing colour to the skin^4^. Melanin is produced by melanocytes in the epidermis and is deposited in melanosomes, which are transferred to adjacent keratinocytes^5^. Melanocytes are also implicated in several other important bioregulatory, metabolic and homeostatic processes, both in the skin and in other organs^5^.

Skin colour varies among different populations and is strongly correlated with latitude due to the variation in UV radiation intensity^6^. Moreover, several selective factors have been implicated in the evolution of human pigmentation towards darker pigmentation in equatorial and tropical regions^2^, including: protection against the harmful effects of UV radiation exposure^7^; protection against folate photolysis^2^,^8^; maintenance of adequate levels vitamin D^9^; and contributing to the skin’s barrier function by optimizing water conservation and improving cutaneous antimicrobial defense^10^.

The colour of unexposed skin (constitutive skin pigmentation) is a complex trait^11^. Indeed, evidence supports that many genes and other interacting factors are involved in determining normal skin pigmentation^12^,^13^. However, candidate-gene and genome-wide association studies (GWAS) have revealed only a few of the total estimated number of genes implicated in the variability of human skin colour^2^,^14^,^15^. Moreover, despite the large differences in skin pigmentation across populations, most genetic association studies of skin colour have been performed in European^16^,^17^ and Asian populations^18^,^19^,^20^. Only a handful of candidate-gene association studies has been performed in African ancestry populations^14^,^21^,^22^,^23^, and one lone GWAS has been carried out in African-European admixed individuals from Cape Verde^24^.

Hispanics/Latinos from Puerto Rico are the result of the admixture of European, African, and Native American ancestry. Specifically, the Native American component derives from the Taínos, the native population of Puerto Rico, which was highly reduced by slavery trade, warfare and diseases^25^, and the European component was introduced by the Spanish settlers^26^,^27^,^28^. Later on, the Spanish brought African slaves who replaced the indigenous population of the island. Therefore, the resulting population has nowadays a predominant European admixture, followed by African ancestry and lower Native American component. Given that performing GWAS in recently admixed African-ancestry populations provides an opportunity to identify novel genes implicated in skin colour variability^14^, we hypothesized that a GWAS in Hispanics/Latinos from Puerto Rico could reveal novel genes contributing to this trait. Herein, we identify genetic variants influencing skin pigmentation in African-admixed individuals analysing 14 million genetic variants across the genome.

## Methods

### Ethics statement

This study has been approved by the institutional review boards of University of California San Francisco and all participant centres. Written informed consent was obtained from all subjects or from their appropriate surrogates for participants under 18 years old. All methods were performed in accordance with the relevant guidelines and regulations for human subject research, in accordance with the Declaration of Helsinki.

### Study populations

Samples from the Genes-environment & Admixture in Latino Americans (GALA II) Study and the Study of African Americans, Asthma, Genes & Environments (SAGE II) were used for the discovery of genetic variants associated with skin colour and replication of results, respectively. The GALA II and SAGE II studies are two independent case-control studies initially conceived for the study of genetic and environmental factors involved in asthma. Both studies used the same protocol and questionnaires to recruit unrelated children aged 8 to 21 years old, but focused on two different racial/ethnic groups: Hispanics/Latinos in GALA II and African Americans in SAGE II. All recruited subjects must have reported that all four grandparents self-identified as Hispanics/Latinos (GALA II) or African Americans (SAGE II). Participants from GALA II with skin colour measurements included in this study were recruited in Puerto Rico, while SAGE II participants were recruited from the San Francisco Bay Area^29^,^30^.

### Skin colour characterization

We used the DSM II ColorMeter (Cortex Technology, Hadsund, Denmark) to measure skin pigmentation in triplicate for each participant along the inner side of each upper arm. Melanin was measured using the melanin index, defined as the inverse of the melanin reflectance measured at 650 nm^24^; lower values of the melanin index correspond to light skin colour, whereas larger values correspond to dark skin colour.

### Genotyping and assessment of genetic ancestry

Genome-wide genotyping data from participants of both studies were obtained by using the Axiom LAT1 array (World Array 4, Affymetrix, Santa Clara, CA, United States), and quality control procedures were performed as described elsewhere^29^,^30^. Genotype data were obtained for the 285 Hispanics/Latinos from Puerto Rico from GALA II and the 373 African Americans from SAGE II who had available skin colour measurement data.

Genetic ancestry was initially assessed by performing a principal components analysis (PCA) using EIGENSOFT^31^. We assessed ancestry structure among Hispanics/Latinos from Puerto Rico and African Americans using the African (YRI) and European (CEU) reference populations from the 1000 Genomes Project (1KGP)^32^, and Native American (NAM) individuals genotyped with the Axiom LAT1 array^29^. Genetic ancestry proportions for each subject were also estimated with an unsupervised model from ADMIXTURE^33^, using the CEU and YRI as parental populations for African Americans, and CEU, YRI and NAM as parental populations for Hispanics/Latinos from Puerto Rico.

### Imputation, association testing and meta-analysis

Genetic variants located in autosomal chromosomes were imputed by means of the Michigan Imputation Server^34^, using SHAPEIT^35^ for haplotype reconstruction, and Minimac3 software for the imputation step^36^. The first release of the Haplotype Reference Consortium (HRC) was used as the reference population^37^.

Association testing with skin colour was performed in Hispanics/Latinos from Puerto Rico by means of the linear Wald test implemented in the software EPACTS 3.2.6^38^, adjusting by the proportions of African and Native American ancestries. The results were then filtered to retain those variants with a minor allele frequency (MAF) ≥1% and Rsq ≥0.3. Variants associated with skin pigmentation in the GALA II discovery sample at a suggestive significance level (*p* ≤ 1 × 10^−5^) were followed up for replication in SAGE II African Americans. Association testing was performed similarly in the replication sample, with the exception that only African ancestry was used to adjust for genetic ancestry.

Results from the discovery and replication samples were meta-analyzed using METASOFT. Random-effects models were applied for single nucleotide polymorphisms (SNPs) showing heterogeneity of effects between studies (Cochran’s Q test *p*-value ≤ 0.05) and fixed effects models for those SNPs without evidence of heterogeneity (Cochran’s Q test *p*-value Q > 0.05)^39^. Genome-wide significance was declared at *p*-value ≤ 5 × 10^−8^.

Chromosomal regions containing variants that were genome-wide significant were plotted for the discovery sample using Locus Zoom 1.1 (ref. 40) based on linkage disequilibrium (LD) data from the 1KGP (GRCh37/hg19 build)^32^. Independence of association signals with skin colour among SNPs located within the same genomic region was assessed by multivariate linear regression analyses conditioned on the most significant SNP of each region using R 3.2.2 (ref. 41).

### Allele frequency distribution assessment of rs6602666

We assessed the distribution of the minor allele frequency of the novel associated variant rs6602666 across different populations. We first used the Geography of Genetic Variants Browser Beta v0.2 to plot allele distributions in African, admixed American, East Asian, European, and South Asian populations from 1KGP Phase III^42^. Given that Native American populations are not represented in the 1KGP dataset, we downloaded publicly available data for 108 Native American individuals described in Lazaridis *et al*.^43^ (7 Bolivian, 12 Karitiana, 18 Mayan, 10 Mixe, 10 Mixtec, 10 Nasoi, 4 Piapoco, 14 Pima, 5 Quechua, 8 Surui, and 10 Zapotec). Allele frequency in those groups was assessed using PLINK^44^.

## Results

### Ancestry composition and skin colour distribution

Our analysis of the ancestral composition using PCA revealed that no individuals were outliers regarding their ancestry composition (Supplementary Fig. S1). As expected, Hispanics/Latinos from Puerto Rico had a larger proportion of European and lower contribution of African and Native American admixture compared with African Americans (Table 1, Supplementary Fig. S2). The replication sample showed a predominant African component and, to a lesser extent, European ancestry (Table 1, Supplementary Fig. S2). Therefore, despite being two African-admixed populations, Hispanics/Latinos from Puerto Rico had significantly smaller proportions of African admixture (22.8% ± 9.5%) compared with African Americans (80.9% ± 10.0%, *p* < 0.001).

**Figure 1:**
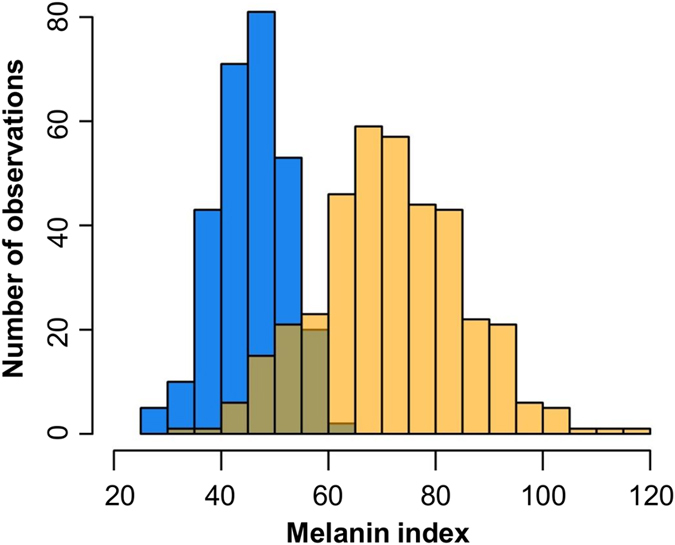
Distribution of melanin index in the discovery and replication samples. The *x*-axis represents the melanin index for Hispanics/Latinos from Puerto Rico (blue) and African American (yellow) samples; the *y*-axis represents the number of observations.

A summary of the descriptive data of the individuals from our study is shown in Table 1. Age average and proportion of males were similar across the discovery and replication samples, and neither of those characteristics was associated with skin pigmentation (*p* > 0.05) and therefore they were not included as covariates in the GWAS. Additionally, Hispanics/Latinos from Puerto Rico had lighter skin (45.8 ± 6.8) than African Americans (71.9 ± 13.5) (*p* < 0.001). Actual distributions of the melanin index for the two populations are shown in Fig. 1.

### Discovery study in Hispanics/Latinos from Puerto Rico

**Figure 2:**
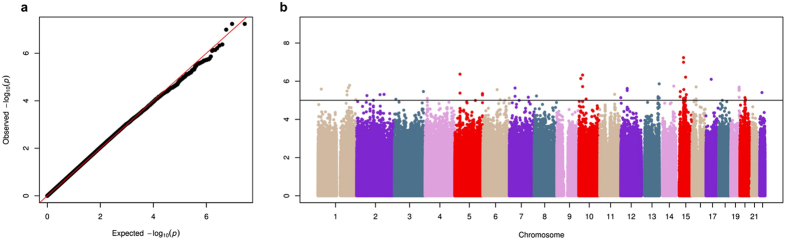
Results of the melanin index GWAS in the discovery stage. (**A**) Quantile-quantile plot showing the observed -log_10_ *p*-values versus the expected -log_10_ *p*-values. (**B**) Manhattan plot of association results (represented as -log_10_ *p*-value on the *y*-axis) along the chromosomes (*x-*axis). The suggestive significance threshold for replication is indicated by the black line (*p* = 1 × 10^−5^).

Association analyses of the 14 million imputed variants with MAF ≥1% in the discovery sample revealed a total of 82 SNPs associated with skin colour at a suggestive significance level (*p*-value ≤ 1 × 10^−5^) (Supplementary Table S1). No major genomic inflation (λ_GC_ = 1.02) was observed in the Q-Q plot (Fig. 2A) and the most significant SNPs were located in chromosomes 5, 10, and 15 (Fig. 2B). The top hit was rs2675345, located within *SLC24A5*, which was near genome-wide significance (*p* = 5.83 × 10^−8^; β for G allele: 3.31, 95% CI: 2.14–4.47).

### Replication of associated variants in African Americans and meta-analysis

Of the 82 SNPs that were significant at a suggestive level in Hispanics/Latinos from Puerto Rico, 77 were followed up for replication in the African American sample, since the remaining five were either monomorphic (three SNPs) or had a MAF <1% (two SNPs). Out of the 77 SNPs, 14 replicated in the African American sample (*p*-value < 0.05) and effect sizes were all in the same direction and of similar magnitude as the discovery sample (Table 2).

The meta-analysis showed evidence of association for nine of the 14 SNPs at a genome-wide significance level (*p* < 5 × 10^−8^) (Table 2). These SNPs are located within three genomic regions. Two regions are already known to contribute to skin colour, including *SLC24A5*^18^,^19^,^20^,^21^,^23^,^45^ and surrounding genes (Supplementary Fig. S3) as well as the *SLC45A2*^18^,^24^,^45^ gene (Supplementary Fig. S4). We also report one novel region described for the first time in the current study, located in the intergenic region of *BEND7* and *PRPF18*. The remaining five SNPs not reaching genome-wide significance were located in three genes *SST-RTP2, ATP8B4*, and *EIF2S2-ASIP*; the two last genes have previously been associated with skin colour-related traits^18^,^23^,^46^,^47^.

Three SNPs within *SLC24A5* showed the strongest meta-analysis association signals: rs1426654 (β for G allele: 4.36, *p* = 2.62 × 10^−14^), rs2675345 (β for G allele: 3.89, *p* = 2.98 × 10^−14^), and rs2470102 (β for G allele: 4.31, *p* = 3.70 × 10^−14^). We also detected two SNPs near *SLC24A5* that were genome-wide significant, one located within *DUT* (rs11637235, β for T allele: −3.83, *p* = 3.34 × 10^−10^) and the other within *MYEF2* (rs8028919, β for A allele: −3.31, *p* = 1.62 × 10^−10^). After including all five SNPs located within or near *SLC24A5* in one common regression model, we determined that the association signal among Hispanics/Latinos from Puerto Rico was driven by the top SNP (rs2675345), as the regression coefficients for the other SNPs were not significant in the common model (Supplementary Table S2).

The second locus with genome-wide significant association with skin colour in the meta-analysis was in *SLC45A2*: rs16891982 (β for G allele: −2.85, *p* = 9.71 × 10^−10^) and rs35397 (β for T allele: −2.66, *p* = 2.05 × 10^−8^). These two SNPs had high LD (*r*^2^ ≥ 0.82) in both populations and their association with skin pigmentation was driven by rs16891982 (a SNP associated with skin colour by previous studies)^18^, given that rs35397 lost significance after performing regression analysis conditioned on rs16891982 (*p* = 0.945) (Supplementary Table S2).

**Figure 3:**
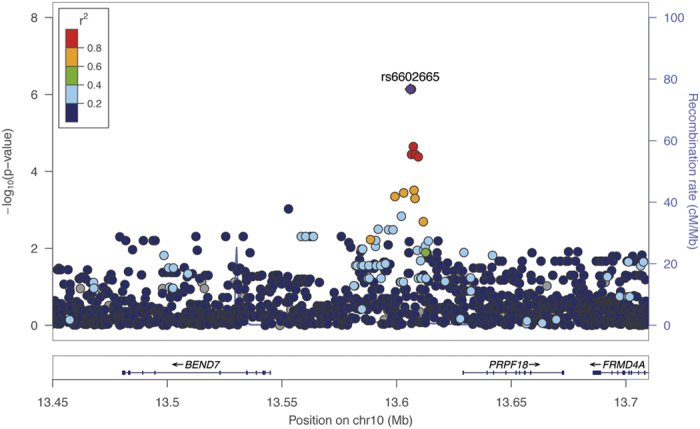
Regional plot of association results in the discovery stage for the *BEND7*/*PRPF18* intergenic region, a novel locus for skin pigmentation. The statistical significance of association results (-log_10_ *p*-value) is represented for each SNP as a dot (*y*-axis) by chromosome position (*x*-axis). The top hit (rs6602665) is represented by a diamond and remaining SNPs are colour coded based on their LD with this SNP, indicated by pairwise *r*^2^ values for American populations of the 1KGP.

Moreover, two SNPs located in the intergenic region of *BEND7* and *PRPF18* (Fig. 3) were associated with skin colour at genome-wide significance in the meta-analysis: rs6602665 (β for C allele: 4.01, *p* = 6.14 × 10^−9^) and rs6602666 (β for G allele: 4.03, *p* = 4.58 × 10^−9^), which showed strong LD in the discovery and replication samples (*r*^2^ = 0.99). These SNPs were more significantly associated with skin colour in Hispanics/Latinos from Puerto Rico (β = 4.72, *p* = 7.27 × 10^−7^ for both SNPs) than in African Americans (β = 3.20, *p* = 1.80 × 10^−3^ and β = 3.14, *p* = 2.34 × 10^−3^ for rs6602666 and rs6602665, respectively). As expected by the high LD between the two SNPs, they represented one association signal in regression analysis when both SNPs were incorporated into the same model (*p* = 0.788 for rs6602666).

**Figure 4:**
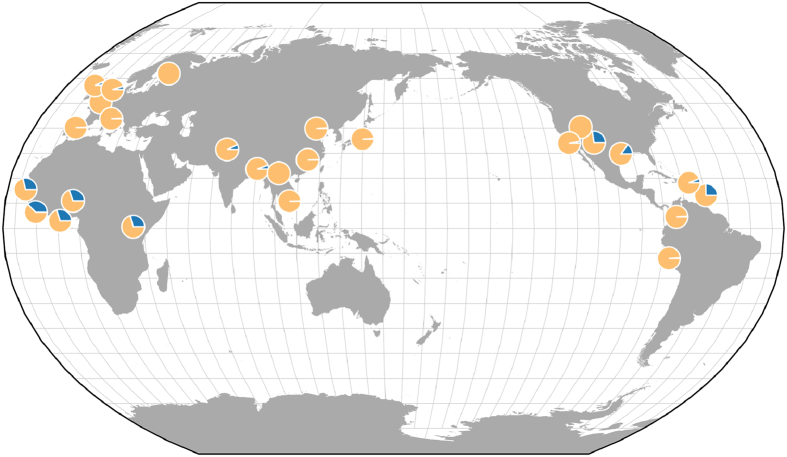
Allele frequency map for rs6602666, the most significant meta-analysis SNP from the intergenic region of *BEND7* and *PRPF18*. Frequency proportions for the effect (G) and non-effect (A) alleles are represented in dark and light gray, respectively. Obtained from the Geography of Genetic Variants Browser Beta v0.2^42^.

The frequency distribution of the G allele of rs6602666 in 1KGP Phase III (Fig. 4) showed that this variant is more prevalent in populations with African ancestry (MAF = 30%) and in South Asians (MAF = 8%), and has a lower frequency in admixed American populations (MAF = 3%). Among admixed American populations, this variant was more prevalent in Puerto Ricans residing in Puerto Rico. In contrast, this variant is almost absent in Europeans and East Asians. An assessment of allele frequency in populations of Native American origin revealed that this variant is monomorphic in the 108 samples from the 11 Native American populations with available data^43^.

## Discussion

In this study, we performed the first GWAS of skin colour in Hispanics/Latinos from Puerto Rico from the GALA II study. After performing genotype imputation and subsequent association testing, we detected 82 suggestive association signals in Hispanics/Latinos, 14 of which replicated at nominal significance in an independent African American sample from the SAGE II study. We identified novel, genome-wide significant associations between skin colour and variants from the *BEND7*/*PRPF18* intergenic region. We also validated the association of five genes already known to contribute to skin colour identified primarily in European populations: two loci with genome-wide significance (*SLC24A5* and *SLC45A2*), and three at a suggestive level (*EIF2S2, ASIP*, and *ATP8B4*). In addition to replicating previously described SNPs^18^,^21^, our results also revealed additional loci within the same region (e.g., rs2675345 from *SLC24A5*).

Among the three most significantly associated gene regions, variants near or within *SLC24A5* showed the strongest association signals. This gene is located in the 15q21.1 chromosomal band and encodes the NCKX5 protein (solute carrier family 24 [sodium/potassium/calcium exchanger], member 5), an intracellular membrane protein whose function has been associated with skin colour and diseases related to skin pigmentation^21^,^48^. The top SNP in our meta-analysis (rs1426654) has been also associated with skin colour in African American and African Caribbean populations in a candidate-gene study^21^, and has broadly replicated across different populations^18^,^19^,^20^,^23^,^24^,^45^.

We also validated the association of *SLC45A2* with skin colour in Hispanics/Latinos from Puerto Rico and African Americans. This gene encodes SLC45A2, which is a transporter highly expressed in the melanosomal membrane of melanocytic cell lines, where it is overexpressed in melanoma cells^49^. *SLC45A2* has been associated with different pigmentary traits (e.g., eye, skin, and hair colour)^18^ and diseases^50^. We confirmed the association of a previously described SNP with normal skin pigmentation (rs16891982 [Phe374Leu]), which was first identified in South Asians^18^ and has since been validated in other populations, including African-admixed individuals^24^,^45^.

Notably, we detected two novel genome-wide significant associations (rs6602665 and rs6602666) in the intergenic region of *BEND7* and *PRPF18* with skin colour. Both variants are located closer to *PRPF18* (approximately 23 kb) than to *BEND7* (approximately 83 kb). The function of the intracellular protein encoded by *BEND7* (BEN domain-containing protein 7) is not extensively known. Nevertheless, it contains the BEN domain, which is involved in transcription regulation throughout recruitment of chromatin remodelling factors and DNA-protein interactions^51^. The other gene located nearest the top SNP of this region, *PRPF18* (pre-mRNA processing factor 18), encodes a splicing factor implicated in pre-mRNA splicing by means of protein-protein interactions^52^. While no skin pigmentation-specific functions have been attributed to any of these two flanking genes, RNA for both genes is expressed in skin regardless of exposure to UV light, with higher levels of expression for *PRPF18*^53^. However, at the protein level, only PRPF18 is expressed in melanocytes and other skin cells^54^.

Interestingly, based on 1KGP data, the rs6602666 G allele (which is associated with darker skin colour) is present in African, South Asian, and admixed American populations, rare in Europeans, and completely absent in Native Americans. Therefore, differences in the proportion of African genetic ancestry may provide a simple explanation as to why this locus has not been detected in previous GWAS, since they were predominantly focused on populations of European descent. This observation underscores the importance and scientific benefit of studying admixed populations, as the inclusion of genetically diverse groups improves statistical power, particularly when genetic variants are rare^55^.

Identification of genes implicated in human skin pigmentation has high anthropological, forensic and biomedical interest^56^,^57^. For example, genes associated with skin colour are also important in regulating vitamin D levels in Caucasian populations^58^. Given that vitamin D deficiency has been implicated with a variety of diseases^59^, and the fact that the majority of circulating vitamin D is derived from photochemical reactions in the skin, genes affecting skin pigmentation could play an indirect role in several diseases^60^. Furthermore, identification of genes involved in controlling melanin levels in the skin could provide new insights regarding the genetics of several types of skin cancer^61^. In fact, some of the skin pigmentation associated loci in our present study, such as *SLC45A2*, have also been associated with protection against basal cell carcinoma, squamous cell carcinoma^62^, and melanoma among Europeans^50^, who are at increased risk of developing skin cancer^60^. Furthermore, another gene associated with skin colour in our study, *ASIP*, has been previously implicated in basal cell carcinoma^16^. Therefore, the novel locus associated with skin pigmentation in the current GWAS might also be relevant for skin cancer susceptibility in African-descent populations. Indeed, data from the National Cancer Institute’s Surveillance, Epidemiology and End Results Program have shown that melanoma incidence is lowest among African Americans (1.0% in females and 1.1% in males), intermediate among Hispanics/Latinos (4.4% in females and 4.8% in males), and highest among non-Hispanic whites (19.4% in females and 32.2% in males)^63^. The lower incidence of melanoma in individuals from populations with darker skin may be attributed to the protective effects of higher melanin levels^64^. Furthermore, there are differences in the prevalence of other types of skin cancer among Hispanics and African Americans, such as basal cell carcinoma, which is the most prevalent skin cancer in Hispanics^65^,^66^ and the second most common skin malignancy in non-Hispanics with African ancestry.

In addition to skin cancer, other diseases of the skin (e.g., vitiligo, psoriasis, or alopecia areata) could be affected by the genetic variants identified in the current study. To date, association studies linking either of the genome-wide significant SNPs (rs6602665 and rs6602666) or their flanking genes (*BEND7* and *PRPF18*) with any of these skin diseases are lacking; future studies should therefore investigate the association of the novel locus with these skin diseases.

Some of the SNPs identified as suggestively associated with skin pigmentation in the current study are located in gene regions previously associated with skin pigmentation, such as *EIF2S2*^46^, *ASIP*^47^, and *ATP8B4*^18^. Nevertheless, we found another suggestive hit near two genes that had not been previously associated with this trait (*SST* and *RTP2*) and deserve further attention in future studies.

Our study has several advantages that should be highlighted: a) skin colour was assessed using skin reflectance spectrometry obtaining a quantitative measure of skin pigmentation, as opposed to many previous studies based on self-reported skin colour; b) skin pigmentation measures were obtained using the same instrument in different recruitment centres participating in the GALA II and SAGE II to reduce possible biases; c) the analysed samples were genotyped with a specific array for African-admixed populations, providing good representation of their genomic variation^67^; d) for the first time in a GWAS of skin colour, the extensive catalogue of genetic variants provided by whole-genome sequencing data from the HRC reference panel was used^37^.

The current study also has some limitations that should be considered. Sample size was relatively limited, yet we had sufficient statistical power in the discovery sample (83%) to detect the association of genetic variants with allele frequencies ≥25% and effects sizes (β) ≥ 3.5. Statistical power was limited for variants with lower allele frequencies and modest effect sizes. Moreover, the differing proportions of African admixture and distinct distributions of skin colour between our study populations point to differences in the genetic architecture of skin colour between our two populations. In fact, a proportion of the SNPs associated with skin colour in Hispanic/Latinos was monomorphic or had a low frequency in African Americans, precluding replication attempts of those variants among African Americans. Given that Hispanics/Latinos from Puerto Rico have lower Native American proportions than other Hispanic/Latino subgroups^29^, our results may not generalize to other Hispanic/Latino groups. Therefore, additional replication should be performed in other populations with different ancestry proportions and skin phototypes.

We measured skin pigmentation using the melanin index. It is possible that our GWAS could have yielded different results had we used alternative methods for measuring melanin, such as pyrrole-2,3,5-trycarboxilic acid (PTCA), aminohydroxyphenylalanine (AHP), or electron paramagnetic resonance spectroscopy (EPR)^68^. Future studies using these alternative methods may provide convergent validity to our results if the associated loci are truly related to the melanogenesis. Finally, clear functional evidence relating the novel locus, *BEND7-PRPF18*, with skin pigmentation has not yet been described. Given the involvement of these genes in pre-mRNA processing and transcription regulation, these loci could be related to melanocytic proliferation. Unfortunately, skin biopsies from the patients in this study are unavailable for performing histologic studies or *in vitro* experiments using epidermal cell cultures. Therefore, the functional role of associated variants will need to be assessed by future studies.

In summary, this GWAS validated the role of *SLC24A5* and *SLC45A2* with skin melanin levels in Hispanics/Latinos from Puerto Rico and African Americans, and identified a novel association of variants in the intergenic region of *BEND7* and *PRPF18* with this trait. Therefore, this study reinforces the advantages and the necessity of analyzing African-admixed populations to identify new loci involved in complex traits.

## Additional Information

**How to cite this article:** Hernandez-Pacheco, N. *et al*. Identification of a novel locus associated with skin colour in African-admixed populations. *Sci. Rep.* **7**, 44548; doi: 10.1038/srep44548 (2017).

**Publisher's note:** Springer Nature remains neutral with regard to jurisdictional claims in published maps and institutional affiliations.

## Supplementary Material

Supplementary Material

## Figures and Tables

**Table 1:** Characteristics of the individuals included in the discovery and replication stages.

Characteristics	Discovery sample	Replication sample	*p*-value
	Hispanics/Latinos (n = 285)	African Americans (n = 373)	
Gender (% male)	47	45	0.515^a^
Mean age (years) (P_25_–P_75_)	15 (13–17)	15 (12–18)	0.590^b^
Mean melanin index ± SD	45.8 ± 6.8	71.9 ± 13.5	<0.001^b^
Mean genetic ancestry (%)			
European	66.8	19.1	<0.001^b^
African	22.8	80.9	<0.001^b^
Native-American	10.4	NA	NA

^a^Pearson χ^2^ test (df = 1; α = 0.05); ^b^Mann-Whitney U test; P_25_: Percentile 25; P_75_: Percentile 75; SD: Standard deviation. NA: Not applicable.

**Table 2:** Melanin index meta-analysis results for suggestively associated SNPs that also nominally replicated.

SNP	Chromosome band	Position	Nearest gene(s)	A1/A2	Discovery sample (n = 285)			Replication sample (n = 373)			Meta-analysis	
					Freq.^a^	β (95%CI) ^b^	p-value	Freq.^a^	β (95%CI) ^b^	p-value	β (95%CI) ^b^	p-value
rs79592764	3q27.3	187398936	SST-RTP2	T/C	0.035	6.38 (1.34 to 9.02)	3.47 × 10^−6^	0.080	3.61 (0.48 to 6.73)	2.43 × 10^−2^	5.23 (3.21 to 7.24)	3.84 × 10^−7^
rs35397	5p13.2	33951116	SLC45A2	T/G	0.511	−2.49 (−3.53 to −1.45)	4.19 × 10^−6^	0.217	−3.29 (−5.35 to −1.24)	1.81 × 10^−3^	−2.66 (−3.58 to −1.73)	2.05 × 10^−8^
rs16891982	5p13.2	33951693	SLC45A2	G/C	0.523	−2.66 (−3.67 to −1.65)	4.27 × 10^−7^	0.189	−3.74 (−5.92 to −1.56)	8.36 × 10^−4^	−2.85 (−3.77 to −1.94)	9.71 × 10^−10^
rs6602665	10p13	13605982	BEND7-PRPF18	C/T	0.079	4.72 (2.89 to 6.54)	7.27 × 10^−7^	0.235	3.14 (1.13 to 5.15)	2.34 × 10^−3^	4.01 (2.66 to 5.36)	6.14 × 10^−9^
rs6602666	10p13	13606490	BEND7-PRPF18	G/A	0.079	4.72 (2.89 to 6.54)	7.27 × 10^−7^	0.237	3.20 (1.20 to 5.19)	1.80 × 10^−3^	4.03 (2.68 to 5.37)	4.58 × 10^−9^
rs2675345	15q21.1	48400199	SLC24A5	G/A	0.277	3.31 (2.14 to 4.47)	5.83 × 10^−8^	0.756	5.57 (3.59 to 7.55)	6.60 × 10^−8^	3.89 (2.89 to 4.89)	2.98 × 10^−14^
rs1426654	15q21.1	48426484	SLC24A5	G/A	0.312	3.06 (1.96 to 4.16)	1.02 × 10^−7^	0.762	5.90 (3.91 to 7.89)	1.31 × 10^−8^	4.36 (1.58 to 7.13) ^c^	2.62 × 10^−14 c^
rs2470102	15q21.1	48433494	SLC24A5	G/A	0.277	3.31 (2.14 to 4.47)	5.83 × 10^−8^	0.756	5.60 (3.63 to 7.57)	5.08 × 10^−8^	4.31 (2.08 to 6.53) ^c^	3.70 × 10^−14 c^
rs8028919	15q21.1	48460188	MYEF2	A/G	0.793	−2.95 (−4.22 to −1.68)	7.99 × 10^−6^	0.385	−3.96 (5.65 to −2.27)	6.15 × 10^−6^	−3.31 (−4.33 to −2.30)	1.62 × 10^−10^
rs11637235	15q21.1	48633153	DUT	T/C	0.509	−2.26 (−3.24 to −1.28)	9.61 × 10^−6^	0.183	−5.71 (−7.93 to −3.48)	7.76 × 10^−7^	−3.83 (−7.20 to −0.46) ^c^	3.34 × 10^−10 c^
rs2899446	15q21.2	50307416	ATP8B4	G/A	0.544	2.28 (1.34 to 3.21)	2.73 × 10^−6^	0.783	2.30 (0.15 to 4.45)	3.67 × 10^−2^	2.28 (1.43 to 3.14)	1.72 × 10^−7^
rs8033655	15q21.2	50308950	ATP8B4	G/A	0.544	2.28 (1.34 to 3.21)	2.73 × 10^−6^	0.784	2.36 (0.21 to 4.51)	3.24 × 10^−2^	2.29 (1.43 to 3.14)	1.54 × 10^−7^
rs7180182	15q21.2	50310295	ATP8B4	G/A	0.544	2.28 (1.34 to 3.21)	2.73 × 10^−6^	0.784	2.36 (0.21 to 4.51)	3.24 × 10^−2^	2.29 (1.43 to 3.14)	1.54 × 10^−7^
rs6142102	20q11.22	32704627	EIF2S2-ASIP	G/C	0.616	2.24 (1.28 to 3.20)	7.28 × 10^−6^	0.420	2.12 (0.42 to 3.82)	1.52 × 10^−2^	2.21 (1.38 to 3.05)	2.22 × 10^−7^

^a^Freq.: Frequency of the effect allele; ^b^Effect size for the effect alleles (additive model); ^c^Random effect model was used since heterogeneity was found between the discovery and replication samples. Abbreviations: A1: Effect allele; A2: Non-effect allele; CI: confidence interval.
