# Supplementary material for: Identification of a novel locus associated with skin colour in African-admixed populations
Source: Sci Rep. 2017 Mar 16;7:44548. doi: 10.1038/srep44548 (PMC5353593; doi:10.1038/srep44548)
Supplement: Supplementary Material [file srep44548-s1.pdf]

SUPPLEMENTARY MATERIAL

Identification of a novel locus associated with skin colour in African-admixed populations

Natalia Hernandez-Pacheco[1,2], Carlos Flores[1,3], Santos Alonso[4], Celeste Eng[5], Angel C.Y. Mak[5], Scott Hunstman[5], Donglei Hu[5], Marquitta J. White[5], Sam S. Oh[5], Kelley Meade[6], Harold J. Farber[7], Pedro C. Avila[8], Denise Serebrisky[9], Shannon M. Thyne[10], Emerita Brigino-Buenaventura[11], William Rodriguez-Cintron[12], Saunak Sen[13], Rajesh Kumar[14], Michael Lenoir[15], Jose R. Rodriguez-Santana[16], Esteban G. Burchard[5,17], Maria Pino-Yanes[1,3]

[1]Research Unit, Hospital Universitario N.S. de Candelaria, Universidad de La Laguna, Santa Cruz de Tenerife, Spain.
[2]Applied Genomics Group (G2A), Section of Genetics, Department of Biochemistry, Microbiology, Cell Biology and Genetics, Universidad de La Laguna, San Cristobal de La Laguna, Tenerife, Spain.
[3]CIBER de Enfermedades Respiratorias, Instituto de Salud Carlos III, Madrid, Spain.
[4]Department of Genetics, Physical Anthropology and Animal Physiology, University of the Basque Country UPV/EHU, Leioa, Bizkaia, Spain.
[5]Department of Medicine, University of California, San Francisco, CA, United States.
[6]Children's Hospital and Research Center Oakland, Oakland, CA, USA.
[7]Department of Pediatrics, Section of Pulmonology, Baylor College of Medicine and Texas Children's Hospital, Houston, TX, USA.
[8]Allergy & ENT Associates, Woodlands, TX, USA.
[9]Pediatric Pulmonary Division, Jacobi Medical Center, Bronx, NY, USA.
[10]Department of Pediatrics, University of California San Francisco, San Francisco General Hospital, San Francisco, CA, USA.
[11]Department of Allergy and Immunology, Kaiser Permanente-Vallejo Medical Center, Vallejo, CA.
[12]Veterans Caribbean Health Care System, San Juan, Puerto Rico.
[13]Division of Biostatistics, Department of Preventive Medicine, UTHSC, Memphis, TN, USA.
[14]Children's Memorial Hospital and the Feinberg School of Medicine, Northwestern University, Chicago, IL, USA.
[15]Bay Area Pediatrics, Oakland, CA, USA.
[16]Centro de Neumología Pediátrica, San Juan, Puerto Rico.
[17]Department of Bioengineering and Therapeutic Sciences, University of California, San Francisco, CA, United States.

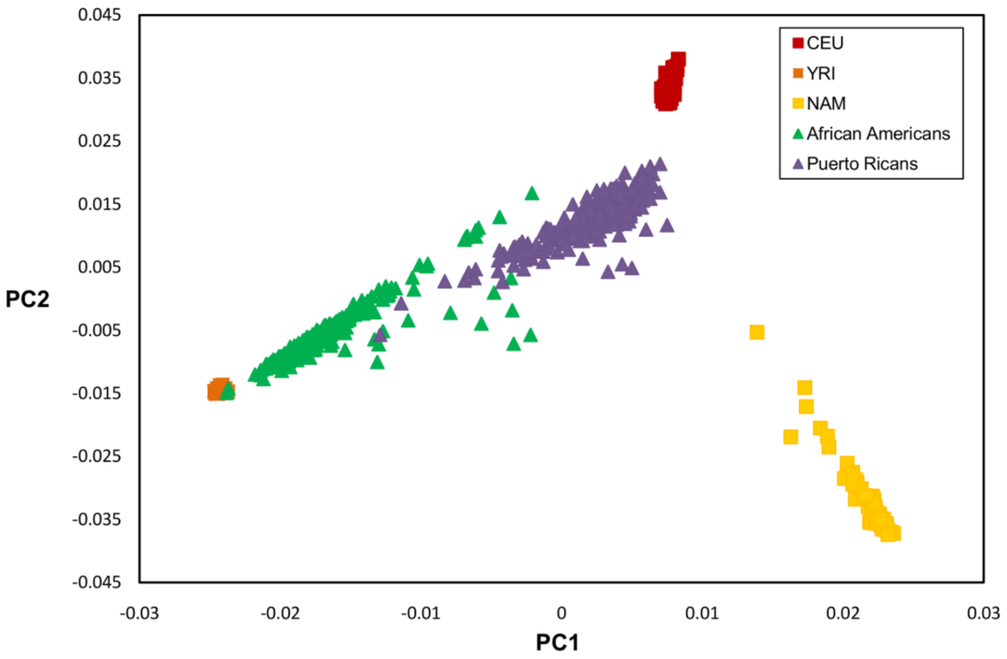

Supplementary Figure S1. PCA plot showing the ancestral composition of the individuals included in the current study, represented by the two first principal components (PC1 and PC2). Reference populations of European (CEU), African (YRI) and Native American (NAM) descent are shown along with the individuals included in discovery (Hispanics/Latinos from Puerto Rico) and replication (African Americans) stages.

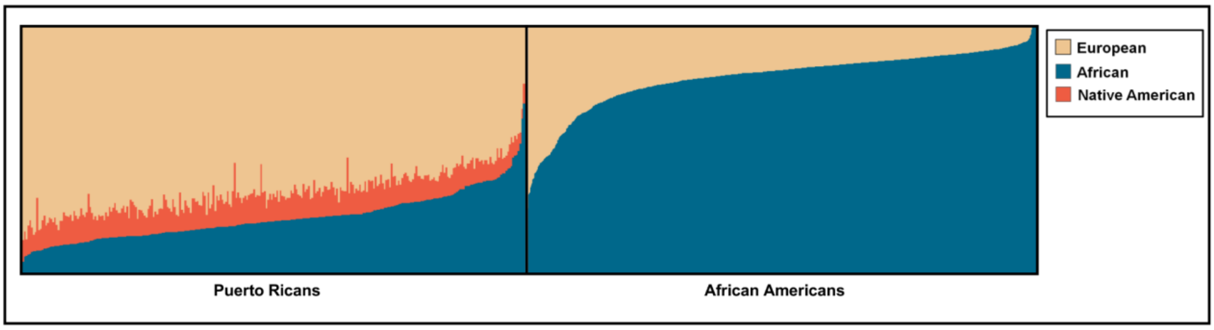

Supplementary Figure S2. Distribution of admixture proportions among Hispanics/Latinos from Puerto Rico and African American individuals. Each subject is represented by one vertical bar and colours show ancestry components. The individuals have been sorted by their proportion of genetic ancestries.

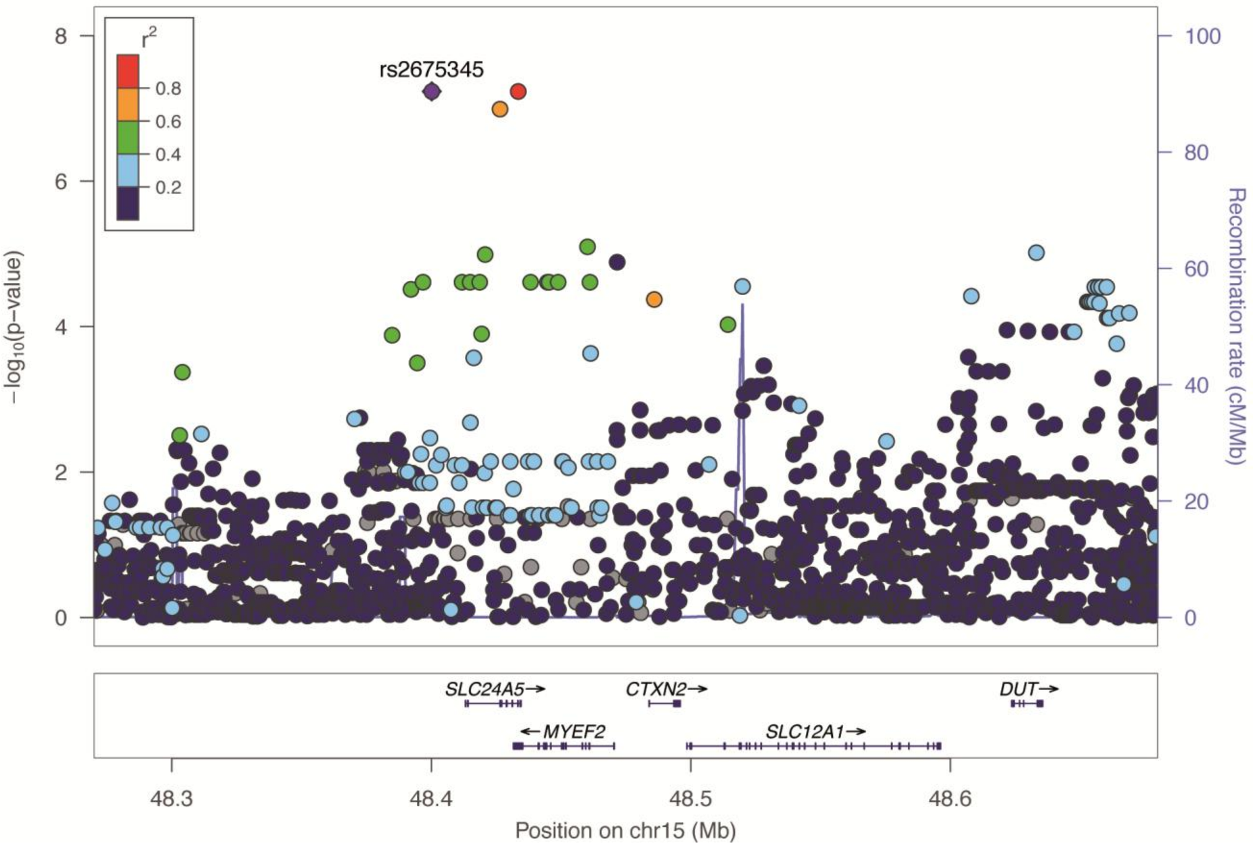

Supplementary Figure S3. Regional plot of association results for *SLC24A5* and surrounding genes in the discovery stage. The transformed probability of association ($-\log_{10}$ *p*-value) is represented for each SNP as a dot by chromosome position. Top hit (rs2675345) is represented with a diamond and the remaining SNPs are colour coded based on their LD with this SNP, indicated by pairwise r^2 values for the Admixed American populations (AMR) from the 1,000 Genomes Project.

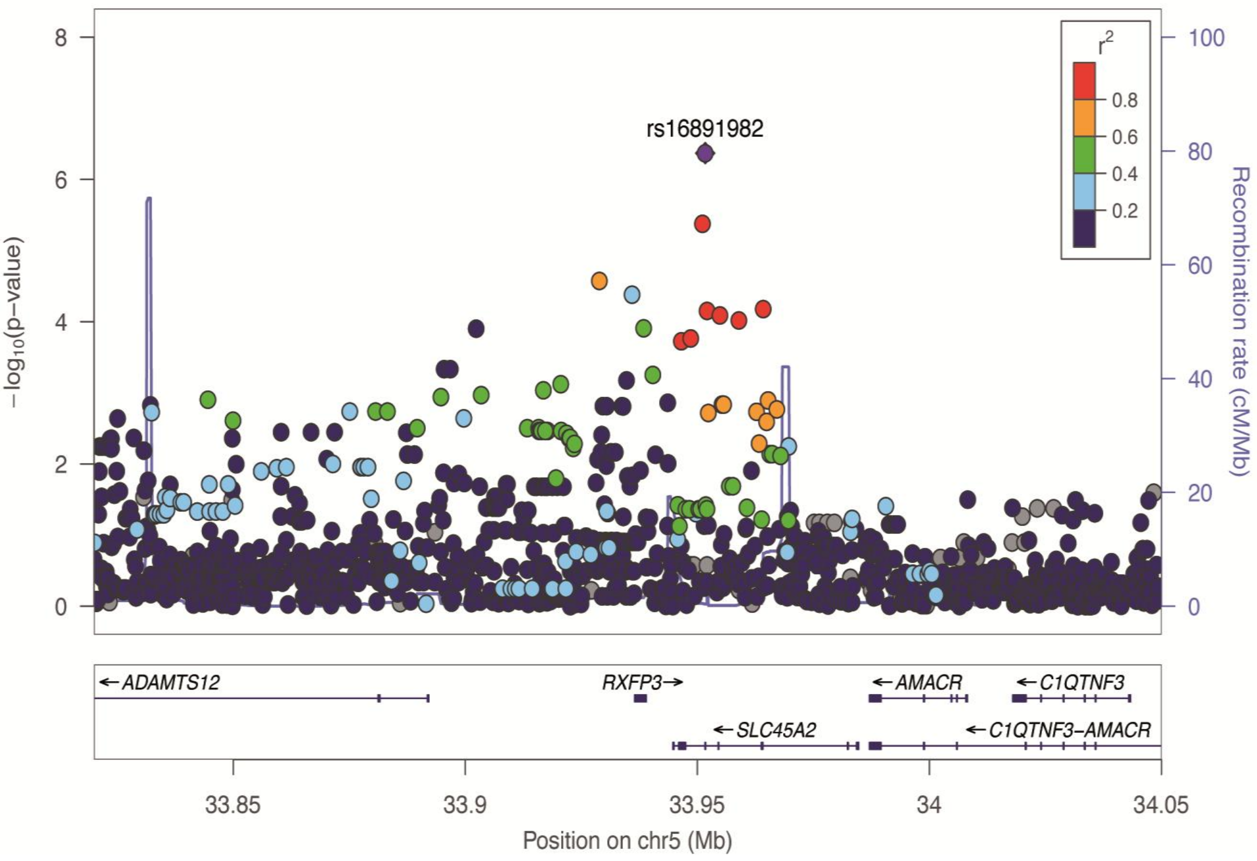

Supplementary Figure S4. Regional plot of association results for *SLC45A2* in the discovery stage. The transformed probability of association results (-log$_{10}$ *p*-value) is represented for each SNP as a dot by chromosome position. The top hit (rs16891982) is depicted by a diamond, and the remaining SNPs are colour coded based on their LD with this SNP, indicated by pairwise r^2 values for the AMR populations of the 1000 Genomes Project.

Supplementary Table S1. Summary of SNPs with *p*-values≤1x10^{-5} for association with melanin index.

SNP	Chromosome band	Position	A1/A2	Freq.(A1)	β (95% CI)	*p*-value
rs1432433	1q31.1	187918615	G/A	0.023	8.02 (4.64 to 11.41)	5.24x10^{-6}
rs77443641	1q31.1	188000243	A/C	0.012	10.94 (6.42 to 15.46)	3.29x10^{-6}
rs115105970	1q31.1	196942660	T/C	0.03	6.84 (4.07 to 9.61)	2.20x10^{-6}
rs112725747	1q32.1	204344757	T/C	0.018	9.46 (5.68 to 13.25)	1.65x10^{-6}
rs6659601	1p36.12	22124820	G/A	0.872	1.69 (0.18 to 3.20)	2.58x10^{-6}
rs76327975	1q44	244364879	G/C	0.023	7.68 (4.35 to 11.01)	9.24x10^{-6}
rs831984	2q12.3	108378250	A/C	0.298	2.59 (1.46 to 3.72)	9.94x10^{-6}
rs831980	2q12.3	108380107	C/G	0.298	2.59 (1.46 to 3.72)	9.94x10^{-6}
rs62170035	2q23.3	151111030	A/C	0.033	6.28 (3.64 to 8.93)	5.07x10^{-6}
rs55767876	2p14	66820715	G/T	0.079	4.51 (2.6 to 6.42)	5.68x10^{-6}
rs66871203	2p14	66821130	G/T	0.079	4.51 (2.6 to 6.42)	5.68x10^{-6}
rs79592764	3q27.3	187398936	T/C	0.035	6.38 (3.74 to 9.02)	3.47x10^{-6}
rs6788400	3p25.3	9886352	G/A	0.089	3.86 (2.19 to 5.52)	8.89x10^{-6}
rs28479566	4p15.33	14776694	T/C	0.035	-6.35 (-9.09 to -3.61)	8.05x10^{-6}
rs340417	5q35.3	178762064	A/C	0.111	-3.86 (-5.48 to -2.24)	4.46x10^{-6}
rs153816	5q35.3	178763605	T/C	0.109	-3.85 (-5.48 to -2.23)	5.24x10^{-6}
rs35397	5p13.2	33951116	T/G	0.511	-2.49 (-3.53 to -1.45)	4.19x10^{-6}
rs16891982	5p13.2	33951693	G/C	0.523	-2.66 (-3.67 to -1.65)	4.27x10^{-7}
rs77437330	6q21	111993747	A/G	0.072	4.45 (2.52 to 6.38)	9.26x10^{-6}
rs73753762	6q15	92184362	G/A	0.014	10.3 (6.08 to 14.52)	2.76x10^{-6}
rs77462788	7q31.32	122042978	C/T	0.021	8.08 (4.63 to 11.54)	6.90x10^{-6}
rs116908038	7q31.32	122168537	C/A	0.021	8.08 (4.63 to 11.54)	6.90x10^{-6}
rs7790204	7p14.2	36684481	C/T	0.549	-2.23 (-3.18 to -1.28)	6.16x10^{-6}
rs7794780	7p14.2	36685144	C/T	0.542	-2.34 (-3.29 to -1.39)	2.28x10^{-6}
rs12541402	8p22	15500967	C/T	0.172	-2.9 (-4.13 to -1.67)	5.93x10^{-6}
rs6602665	10p13	13605982	C/T	0.079	4.72 (2.89 to 6.54)	7.27x10^{-7}
rs6602666	10p13	13606490	G/A	0.079	4.72 (2.89 to 6.54)	7.27x10^{-7}
rs151165649	10p12.1	25207241	A/G	0.019	9.42 (5.84 to 13.00)	4.75x10^{-7}
rs141664730	10p12.1	25338228	C/G	0.016	9.83 (5.87 to 13.79)	1.91x10^{-6}

rs149207584	10p12.1	25339373	C/T	0.016	9.83 (5.87 to 13.79)	$1.91x10^{-6}$
rs111256285	10q11.22	47632167	G/A	0.016	-8.43 (-12.08 to -4.79	$8.61x10^{-6}$
rs7477798	10q11.22	47632478	C/A	0.016	-8.43 (-12.08 to -4.79	$8.61x10^{-6}$
rs115019323	11q21	95895552	A/G	0.025	7.12 (4.13 to 10.12)	$4.85x10^{-6}$
rs144848699	11q21	95895953	C/T	0.025	7.12 (4.13 to 10.12)	$4.85x10^{-6}$
rs1902910	12q12	41778982	G/A	0.104	3.88 (2.3 to 5.46)	$2.45x10^{-6}$
rs17129378	12q12	41861386	C/T	0.104	3.99 (2.35 to 5.63)	$3.05x10^{-6}$
rs17578886	12q12	41862536	G/A	0.084	4.33 (2.57 to 6.09)	$2.40x10^{-6}$
rs10849455	12p13.33	785468	G/T	0.811	2.94 (1.68 to 4.21)	$7.29x10^{-6}$
rs138891280	13q33.2	106722485	G/A	0.021	8.06 (4.59 to 11.52)	$7.85x10^{-6}$
rs188019015	13q33.2	106743244	T/C	0.016	9.33 (5.35 to 13.31)	$6.51x10^{-6}$
rs146078872	13q33.2	106745894	G/A	0.016	9.33 (5.35 to 13.31)	$6.51x10^{-6}$
rs114396339	13q33.2	106746964	T/A	0.016	9.33 (5.35 to 13.31)	$6.51x10^{-6}$
rs727752	13q33.2	106754594	A/C	0.016	9.33 (5.35 to 13.31)	$6.51x10^{-6}$
rs2016565	13q33.2	106754788	C/T	0.016	9.33 (5.35 to 13.31)	$6.51x10^{-6}$
rs115940594	13q33.2	106754945	T/C	0.016	9.33 (5.35 to 13.31)	$6.51x10^{-6}$
rs3024737	13q34	113819785	G/A	0.012	11.26 (6.79 to 15.73)	$1.37x10^{-6}$
rs7326155	13q34	114460362	C/T	0.014	9.78 (5.58 to 13.99)	$7.59x10^{-6}$
rs10133804	14q32.12	92866905	C/T	0.011	1.44 (-3.58 to 6.45)	$1.81x10^{-6}$
rs11858919	15q12	26599133	T/C	0.025	7.59 (4.35 to 10.83)	$6.48x10^{-6}$
rs2675345	15q21.1	48400199	G/A	0.277	3.31 (2.14 to 4.47)	$5.83x10^{-6}$
rs1426654	15q21.1	48426484	G/A	0.312	3.06 (1.96 to 4.16)	$1.02x10^{-7}$
rs2470102	15q21.1	48433494	G/A	0.277	3.31 (2.14 to 4.47)	$5.83x10^{-8}$
rs8028919	15q21.1	48460188	A/G	0.793	-2.95 (-4.22 to -1.68)	$7.99x10^{-6}$
rs11637235	15q21.1	48633153	T/C	0.509	-2.26 (-3.24 to -1.28)	$9.61x10^{-6}$
rs2899446	15q21.2	50307416	G/A	0.544	2.28 (1.34 to 3.21)	$2.73x10^{-6}$
rs8033655	15q21.2	50308950	G/A	0.544	2.28 (1.34 to 3.21)	$2.73x10^{-6}$
rs7180182	15q21.2	50310295	G/A	0.544	2.28 (1.34 to 3.21)	$2.73x10^{-6}$
rs4580097	15q21.2	50315253	A/G	0.544	2.28 (1.34 to 3.21)	$2.73x10^{-6}$
rs28753701	15q22.1	59175467	T/C	0.963	-6.16 (-8.83 to -3.49)	$9.23x10^{-6}$
rs77045588	15q22.2	61136327	A/C	0.012	10.52 (6.01 to 15.02)	$7.23x10^{-6}$
rs78604138	15q22.2	61144845	G/A	0.012	10.52 (6.01 to 15.02)	$7.23x10^{-6}$

rs79617268	15q22.2	61145173	G/C	0.012	10.52 (6.01 to 15.02)	$7.23x10^{-6}$
rs532282237	15q22.2	61817211	C/T	0.011	12.51 (7.7 to 17.31)	$6.14x10^{-7}$
rs61310892	15q22.31	66319806	A/G	0.014	10.02 (5.8 to 14.25)	$5.04x10^{-6}$
rs7200304	16p13.12	13844197	C/T	0.916	4.2 (2.37 to 6.03)	$9.99x10^{-6}$
rs7193564	16p13.12	13844299	A/G	0.916	4.2 (2.37 to 6.03)	$9.99x10^{-6}$
rs7200773	16p13.12	13845690	A/G	0.916	4.2 (2.37 to 6.03)	$9.99x10^{-6}$
rs4141382	16p13.12	13845726	A/T	0.916	4.2 (2.37 to 6.03)	$9.99x10^{-6}$
rs7185574	16p13.12	13846274	T/A	0.916	4.2 (2.37 to 6.03)	$9.99x10^{-6}$
rs9926165	16p12.1	26958800	T/C	0.602	2.32 (1.32 to 3.32)	$8.62x10^{-6}$
rs9926268	16p12.1	26958967	T/C	0.600	2.32 (1.32 to 3.33)	$8.48x10^{-6}$
rs62029775	16p12.1	26959698	T/C	0.609	2.48 (1.48 to 3.48)	$1.99x10^{-6}$
rs117307642	17q12	33823098	T/C	0.042	6.42 (3.93 to 8.91)	$7.94x10^{-7}$
rs62143248	19q13.42	54364168	T/C	0.079	-4.72 (-6.63 to -2.81)	$2.04x10^{-6}$
rs62143250	19q13.42	54365667	A/G	0.079	-4.72 (-6.63 to -2.81)	$2.04x10^{-6}$
rs8102993	19q13.42	54366811	T/C	0.079	-4.68 (-6.59 to -2.78)	$2.35x10^{-6}$
rs11882947	19q13.42	54368893	A/G	0.074	-4.77 (-6.73 to -2.81)	$3.00x10^{-6}$
rs16985221	19q13.42	54374041	T/C	0.1	-3.92 (-5.6 to -2.24)	$6.98x10^{-6}$
rs62143251	19q13.42	54374699	A/G	0.096	-4.14 (-5.83 to -2.45)	$2.55x10^{-6}$
rs6142102	20q11.22	32704627	G/C	0.616	2.24 (1.28 to 3.2)	$7.28x10^{-6}$
rs755107	20q11.23	36662831	G/A	0.039	-5.5 (-7.88 to -3.11)	$9.36x10^{-6}$
rs201429679	22q12.2	31113081	T/G	0.032	6.91 (4.04 to 9.79)	$3.92x10^{-6}$

Supplementary Table S2. Results of the regression models in Hispanics/Latinos from Puerto Rico conditioned on the top hit of each chromosome region with genome-wide significant signals in the meta-analysis.

**Genomic region**	**SNP**	**Regression conditioned on**	**Original *p*-value of the SNP**	***p*-value conditional regression**
SLC24A5, MYEF2, DUT, ATP8B4	rs1426654	rs2675345	1.02×10^{-7}	0.463
	rs2470102		5.83×10^{-8}	NA [a]
	rs8028919		7.99×10^{-6}	0.826
	rs11637235		9.61×10^{-6}	0.112
SLC45A2	rs35397	rs16891982	4.19×10^{-6}	0.945
BEND7-PRPF18	rs6602666	rs6602665	7.27×10^{-7}	0.788

[a] The regression was not applicable due to the existence of co-linearity among rs2470102 and rs2675345 and high LD (R^2=1).
